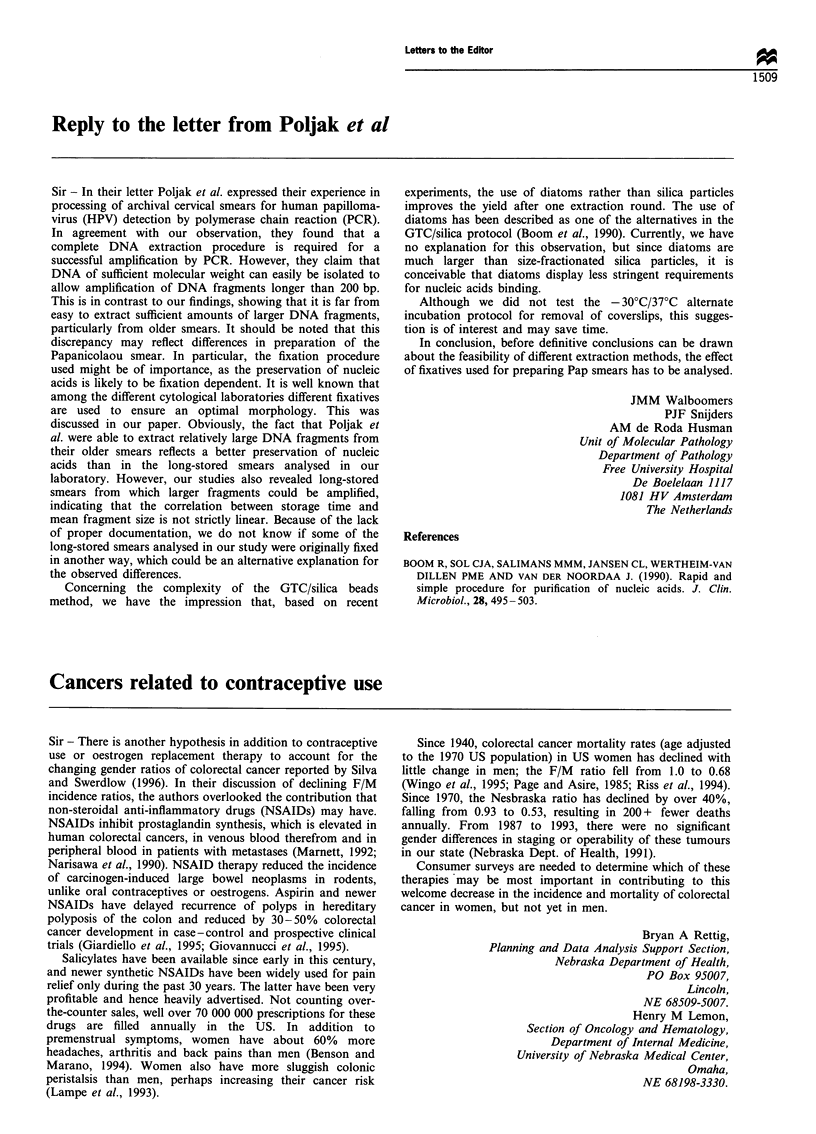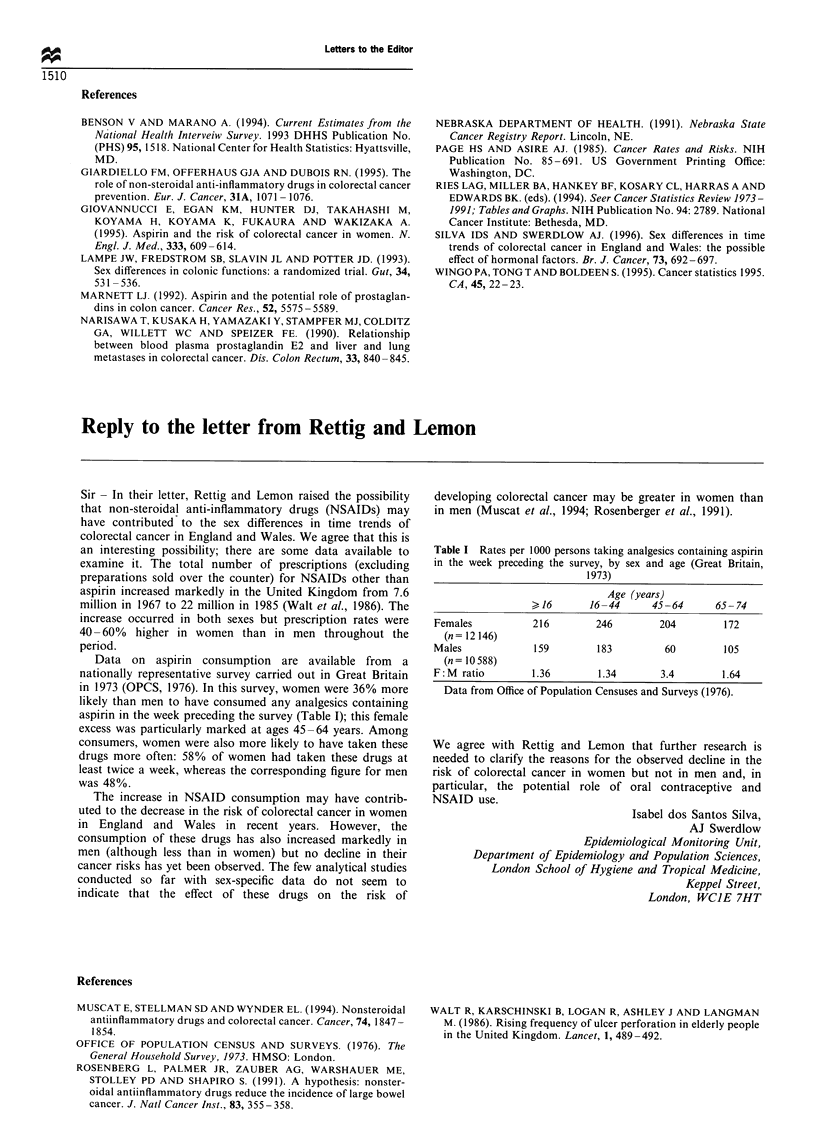# Cancers related to contraceptive use.

**DOI:** 10.1038/bjc.1996.578

**Published:** 1996-11

**Authors:** B. A. Rettig, H. M. Lemon


### References

BOOM R, SOL CJA, SALIMANS MMM, JANSEN CL, WERTHEIM-VAN DILLEN PME AND VAN DER NOORDAA J. (1990). Rapid and simple procedure for purification of nucleic acids. *J. Clin. Microbiol.*, **28**, 495–503.

### Cancers related to contraceptive use

Sir – There is another hypothesis in addition to contraceptive use or oestrogen replacement therapy to account for the changing gender ratios of colorectal cancer reported by Silva and Swerdlow (1996). In their discussion of declining F/M incidence ratios, the authors overlooked the contribution that non-steroidal anti-inflammatory drugs (NSAIDs) may have. NSAIDs inhibit prostaglandin synthesis, which is elevated in human colorectal cancers, in venous blood therefrom and in peripheral blood in patients with metastases (Marnett, 1992; Narisawa *et al.*, 1990). NSAID therapy reduced the incidence of carcinogen-induced large bowel neoplasms in rodents, unlike oral contraceptives or oestrogens. Aspirin and newer NSAIDs have delayed recurrence of polyps in hereditary polyposis of the colon and reduced by 30–50% colorectal cancer development in case–control and prospective clinical trials (Giardiello *et al.*, 1995; Giovannucci *et al.*, 1995).

Salicylates have been available since early in this century, and newer synthetic NSAIDs have been widely used for pain relief only during the past 30 years. The latter have been very profitable and hence heavily advertised. Not counting over-the-counter sales, well over 70 000 000 prescriptions for these drugs are filled annually in the US. In addition to premenstrual symptoms, women have about 60% more headaches, arthritis and back pains than men (Benson and Marano, 1994). Women also have more sluggish colonic peristalsis than men, perhaps increasing their cancer risk (Lampe *et al.*, 1993).

Since 1940, colorectal cancer mortality rates (age adjusted to the 1970 US population) in US women has declined with little change in men; the F/M ratio fell from 1.0 to 0.68 (Wingo *et al.*, 1995; Page and Asire, 1985; Riss *et al.*, 1994). Since 1970, the Nesbraska ratio has declined by over 40%, falling from 0.93 to 0.53, resulting in 200+ fewer deaths annually. From 1987 to 1993, there were no significant gender differences in staging or operability of these tumours in our state (Nebraska Dept. of Health, 1991).

Consumer surveys are needed to determine which of these therapies may be most important in contributing to this welcome decrease in the incidence and mortality of colorectal cancer in women, but not yet in men.

Bryan A Rettig,
*Planning and Data Analysis Support Section,*
*Nebraska Department of Health,*
*PO Box 95007,*
*Lincoln,*
*NE 68509-5007.*
Henry M Lemon,
*Section of Oncology and Hematology,*
*Department of Internal Medicine,*
*University of Nebraska Medical Center,*
*Omaha,*
*NE 68198-3330.*

**References**

BENSON V AND MARANO A. (1994). *Current Estimates from the National Health Interveiw Survey*. 1993 DHHS Publication No. (PHS) **95,** 1518. National Center for Health Statistics: Hyattsville, MD.
GIARDIELLO FM, OFFERHAUS GJA AND DUBOIS RN. (1995). The role of non-steroidal anti-inflammatory drugs in colorectal cancer prevention. *Eur. J. Cancer*, **31A**, 1071-1076.
GIOVANNUCCI E, EGAN KM, HUNTER DJ, TAKAHASHI M, KOYAMA H, KOYAMA K, FUKAURA AND WAKIZAKA A. (1995). Aspirin and the risk of colorectal cancer in women. *N. Engl. J. Med.*, **333,** 609-614.
LAMPE JW, FREDSTROM SB, SLAVIN JL AND POTTER JD. (1993). Sex differences in colonic functions: a randomized trial. *Gut*, **34,** 531-536.
MARNETT LJ. (1992). Aspirin and the potential role of prostaglandins in colon cancer. *Cancer Res.*, **52,** 5575-5589.
NARISAWA T, KUSAKA H, YAMAZAKI Y, STAMPFER MJ, COLDITZ GA, WILLETT WC AND SPEIZER FE. (1990). Relationship between blood plasma prostaglandin E2 and liver and lung metastases in colorectal cancer. *Dis. Colon Rectum*, **33,** 840-845.
NEBRASKA DEPARTMENT OF HEALTH. (1991). *Nebraska State Cancer Registry Report*. Lincoln, NE.
PAGE HS AND ASIRE AJ. (1985). *Cancer Rates and Risks*. NIH Publication No. 85-691. US Government Printing Office: Washington, DC.
RIES LAG, MILLER BA, HANKEY BF, KOSARY CL, HARRAS A AND EDWARDS BK. (eds). (1994). *Seer Cancer Statistics Review 1973-1991; Tables and Graphs*. NIH Publication No. 94: 2789. National Cancer Institute: Bethesda, MD.
SILVA IDS AND SWERDLOW AJ. (1996). Sex differences in time trends of colorectal cancer in England and Wales: the possible effect of hormonal factors. *Br. J. Cancer*, **73,** 692-697.
WINGO PA, TONG T AND BOLDEEN S. (1995). Cancer statistics 1995. *CA*, **45,** 22-23.